# GLUT3 is induced during epithelial-mesenchymal transition and promotes tumor cell proliferation in non-small cell lung cancer

**DOI:** 10.1186/2049-3002-2-11

**Published:** 2014-07-29

**Authors:** Mark Masin, Jessica Vazquez, Simona Rossi, Svenja Groeneveld, Natasha Samson, Petra C Schwalie, Bart Deplancke, Laura E Frawley, Jérôme Gouttenoire, Darius Moradpour, Trudy G Oliver, Etienne Meylan

**Affiliations:** 1 Swiss Institute for Experimental Cancer Research, School of Life Sciences, Ecole Polytechnique Fédérale de Lausanne, Lausanne 1015, Switzerland; 2 Bioinformatics Core Facility, Swiss Institute of Bioinformatics, Lausanne 1015, Switzerland; 3 Institute of Bioengineering, School of Life Sciences, Ecole Polytechnique Fédérale de Lausanne, Lausanne 1015, Switzerland; 4 Koch Institute for Integrative Cancer Research, Massachusetts Institute of Technology, Cambridge, MA 02139, USA; 5 Division of Gastroenterology and Hepatology, Centre Hospitalier Universitaire Vaudois, University of Lausanne, Lausanne 1011, Switzerland; 6 Huntsman Cancer Institute, University of Utah, Salt Lake City, UT 84112, USA

**Keywords:** Epithelial-mesenchymal transition, Glucose transporter, GLUT3, Non-small cell lung cancer, SLC2A3, ZEB1

## Abstract

**Background:**

Alterations in glucose metabolism and epithelial-mesenchymal transition (EMT) constitute two important characteristics of carcinoma progression toward invasive cancer. Despite an extensive characterization of each of them separately, the links between EMT and glucose metabolism of tumor cells remain elusive. Here we show that the neuronal glucose transporter GLUT3 contributes to glucose uptake and proliferation of lung tumor cells that have undergone an EMT.

**Results:**

Using a panel of human non-small cell lung cancer (NSCLC) cell lines, we demonstrate that GLUT3 is strongly expressed in mesenchymal, but not epithelial cells, a finding corroborated in hepatoma cells. Furthermore, we identify that ZEB1 binds to the *GLUT3* gene to activate transcription. Importantly, inhibiting GLUT3 expression reduces glucose import and the proliferation of mesenchymal lung tumor cells, whereas ectopic expression in epithelial cells sustains proliferation in low glucose. Using a large microarray data collection of human NSCLCs, we determine that *GLUT3* expression correlates with EMT markers and is prognostic of poor overall survival.

**Conclusions:**

Altogether, our results reveal that *GLUT3* is a transcriptional target of ZEB1 and that this glucose transporter plays an important role in lung cancer, when tumor cells loose their epithelial characteristics to become more invasive. Moreover, these findings emphasize the development of GLUT3 inhibitory drugs as a targeted therapy for the treatment of patients with poorly differentiated tumors.

## Background

The reprogramming of energy metabolism constitutes one of the hallmarks of cancer [1]. In order to build the necessary biomass required for proliferation, tumor cells increase their glucose consumption [2]. Glucose transporters of the GLUT (*SLC2A*) family are at the first step of cellular glucose utilization, mediating glucose entry by facilitative diffusion. The GLUT family is composed of 14 members that transport glucose or other substrates in different tissues, with different efficiencies [3]. In various tumor types, an increased expression of GLUT1 has been reported [4]. Furthermore, GLUT1 levels are higher in colorectal cancer cell lines harboring *KRAS* or *BRAF* mutations compared with isogenic clones and confer cell survival properties in low-glucose conditions [5]. Interestingly, in an oncogenic Kras(G12D)-dependent mouse model of pancreatic ductal adenocarcinoma (PDAC), oncogene withdrawal led to reduced *Glut1* expression and glucose uptake [6]. In contrast to GLUT1, little is known about the regulation and function of another glucose transporter, GLUT3, in cancer. GLUT3 was originally referred to as the neuronal GLUT [7]; with a high affinity for glucose (*K*_M_ approximately 1.5 mM) and the highest calculated turnover number of all glucose transporters, it ensures efficient glucose uptake by neurons of the central nervous system. With the exception of neurons and a few hematopoietic cell types, GLUT3 is lowly or not expressed in most organs of healthy adults. However, pathological GLUT3 expression has been reported in gastric, testicular, ovarian, and non-small cell lung cancer (NSCLC) [8,9]. Recently, GLUT3 was shown to be highly expressed in glioblastoma and to promote the growth of brain tumor initiating cells [10]. *GLUT3* was also reported to be a transcriptional target of NF-κB and HMGA1, in mouse embryonic fibroblasts and human colorectal tumor cells, respectively [11,12]. However, the regulation and contribution of GLUT3 to lung tumor progression remain unknown.

The epithelial-mesenchymal transition (EMT) is a process that occurs early in embryonic development, notably during gastrulation, in which epithelial cells undergo cytoskeletal changes and lose cell-cell contacts to gain mesenchymal traits and become more motile [13]. During carcinoma progression, pathological EMT occurs to promote tumor cell invasion and metastasis. One of the essential steps in the EMT process is the loss of E-cadherin, an adherens junction protein that maintains cell-cell adhesion and epithelial tissue integrity. Mechanistically, the EMT process can be triggered by different transcription factors that include TWIST, ZEB1, ZEB2, SNAIL, and SLUG [14]. In our study, we discovered a strong association between the EMT program and the induction of the glucose transporter GLUT3 in NSCLC and extended this observation to tumor cells from another cancer type, hepatocellular carcinoma (HCC). We demonstrate that *GLUT3* is a direct transcriptional target of ZEB1. We further show that GLUT3 expression contributes to proliferation of lung tumor cells and is an independent prognostic factor of poor overall survival in NSCLC.

## Methods

### Plasmid constructs

Human *GLUT3*, mouse *Snail*, and mouse *Zeb1* cDNAs, purchased from Thermo Scientific (Waltham, MA, USA; MHS1010-7429646, MMM1013-7510291, and MMM1013-99828709, respectively), were amplified by polymerase chain reaction (PCR) using forward 5′-CTCATCGATGCCACCATGGGGACACAGAAGGT-3′ and reverse 5′-CTCCCCGGGTTAGACATTGGTGGTGG-3′ (*GLUT3*), forward 5′-CTCATCGATGCCACCATGCCGCGCTCCTTC-3′ and reverse 5′-CTCGAATTCTCAGCGAGGGCCTCC-3′ (*Snail*), and forward 5′-CTCATCGATGCCACCATGGCGGATGGCCCC-3′ and reverse 5′-CTCGAATTCCTAAGCTTCATTTGT-3′ (*Zeb1*) oligos. The PCR products were digested with *Cla*I and *Xma*I (*GLUT3*) or *Cla*I and *Eco*RI (*Snail* and *Zeb1*) and cloned into identically digested pRDI292-CMV lentiviral vector (gift of D. Trono, EPFL, Lausanne). Genomic DNA was used to amplify by PCR (a) an approximately 1,000-bp region ending close to the transcription start site of human *SLC2A3* (gene encoding GLUT3), with the oligos forward 5′-CTCGAGCTCGAGACTAGCAGAAAGTG-3′ and reverse 5′-CTCCTCGAGCGACAAGCCCCCAGCCCCACCCCACCCCACCCCACCCCCCTGAAGCAA-3′, or (b) a region containing the *SLC2A3* intron 2 sequence, with the oligos forward 5′-CTCGAGCTCACTGGGGTCATCAATGCTCC-3′ and reverse 5′-CTCCTCGAGGGTTGGTGGAAGAACAGAC-3′. After *Sac*I and *Xho*I digestion, the fragments were cloned into an identically digested luciferase reporter plasmid containing a minimal promoter (kindly provided by J. Huelsken, EPFL, Lausanne) to generate prom.-LUC or int.2-LUC constructs, respectively. Deletion of the E-box-like motif CACCTC from the intron 2 sequence was achieved by site-directed mutagenesis, using oligos forward 5′-CCACTCTTTATAGTGATGCACATCCTG-3′ and reverse 5′-CATCACTATAAAGAGTGGGAGGAAGAAC-3′, combined with the oligos indicated above in (b). shRNAs specific to *GLUT3* were either from Thermo Scientific (TRCN0000042880) or designed using the pSICOLIGOMAKER 1.5 program (created by A. Ventura, Memorial Sloan-Kettering Cancer Center, New York). In the latter case, forward 5′-TGCAAGGATGTCACAAGAAATTCAAGAGATTTCTTGTGACATCCTTGCTTTTTTC-3′ and reverse 5′-TCGAGAAAAAAGCAAGGATGTCACAAGAAATCTCTTGAATTTCTTGTGACATCCTTGCA-3′ oligos were annealed and ligated into a pSicoR lentiviral vector. The fidelity of all the PCR amplifications and oligo syntheses was confirmed by sequencing. Control pLKO.1 was from Thermo Scientific.

### Immunoprecipitation

Cells (8 × 10^7^ per immunoprecipitation) were lysed in NP-40 buffer (0.2% NP-40, 150 mM NaCl, 20 mM Tris pH 8.0, 10 mM EDTA) containing a protease inhibitor cocktail (complete, Roche, Basel, Switzerland) and 1 mM Na_3_VO_4_ for 15 min on ice, followed by three quick steps of freezing in liquid N_2_ and thawing at 37°C. Pre-clearing was achieved using sepharose-6B (Sigma-Aldrich, St. Louis, MO, USA) for 60 min at 4°C on a rotating wheel. Immunoprecipitation was performed using a 1:1 mixture of sepharose-6B and protein-G sepharose (Sigma-Aldrich), together with 2 μg control or ZEB1 antibody, overnight at 4°C on a rotating wheel. After four steps of washing in lysis buffer, sample buffer was added, and the samples were boiled and loaded on a polyacrylamide gel for electrophoresis followed by Western blot.

### Western blotting

Except when used for immunoprecipitation, cells were lysed in RIPA buffer (20 mM Tris pH 8, 50 mM NaCl, 0.5% Na-deoxycholate, 0.1% SDS, 1 mM Na_3_VO_4_, protease inhibitor cocktail (complete, Roche)) for 5 min on ice. Proteins were loaded on 8% or 10% polyacrylamide gels for electrophoresis (150 V, 1 h). Transfer was performed on PVDF membranes (100 V, 1 h).

### Cell culture conditions

The human embryonic kidney (HEK) 293 T cells and the human hepatoma cell lines, which include HLF, HLE, Huh-1 (all three kindly provided by K. Morikawa, Showa University, Tokyo, Japan), Huh-7, and Hep3B, were grown in DMEM. The human non-small cell lung cancer cell lines include A549, SW1573, NCI-H23, NCI-H2122, NCI-H441, NCI-H460, NCI-H727, NCI-H2009, NCI-H1944, and Calu-6; they were all obtained from ATCC (Manassas, VA, USA) and all grown in RPMI. All cell media were supplemented with 10% FBS. For the experiments with high or low glucose, cells were cultured for 4 days in normal RPMI (containing 11 mM glucose), supplemented by 10% FBS, or with RPMI without glucose, supplemented by 10% FBS, resulting in a final concentration of 11.5 mM and 0.5 mM glucose, respectively. After 2 days of culture, the medium was replaced with fresh medium to maintain the initial concentrations of glucose.

### Cell counting

To measure cell number, a trypan blue exclusion assay was performed. Cells were mixed with trypan blue, and the number of live, dead, and total cells was counted on an automated cell counter (Countess, Life Technologies, Carlsbad, CA, USA).

### 2-Deoxy-d-[^3^H]glucose uptake

Cells (1 × 10^6^ per well, seeded the day before the experiment) were cultured on six-well plates. 2-Deoxyglucose uptake assays were performed as described previously [15]. Radioactivity was determined by scintillation counting. For sample normalization, protein concentration was measured by BCA protein assay.

### Transfection

siRNAs were from Life Technologies. siRNA transfection was performed with RNAiMAX transfection reagent (Life Technologies), according to the manufacturer’s instructions. For each siRNA, 10 nM was used. cDNA transfection of 293 T cells was performed with Lipofectamine 2000 (Life Technologies), according to the manufacturer’s instructions. For each well of a 12-well plate, a mix of 125 ng Renilla-Luciferase (phRL-TK, Promega, Madison, WI, USA) and 1.6 μg GLUT3-Luciferase (promoter, intron 2-WT or intron 2-Δ-CACCTC) was used. Twenty-four hours after transfection, cells were lysed and a luciferase reporter assay was performed (Dual-Luciferase Reporter Assay System, Promega). The reported luciferase activity was the ratio between Firefly (GLUT3 construct)-Luciferase and Renilla-Luciferase, which was then normalized to 1 to get fold activities. For double transfection, 293 T cells were transfected first with siRNAs and re-transfected 72 h later with plasmids, 24 h prior to lysis.

### Anchorage-independent growth assays

Cells (9 × 10^4^) were plated in triplicates in six-well plates in 0.4% agar in RPMI on top of a layer of 0.8% agar with RPMI. Cells were allowed to grow at 37°C for 3 weeks. Colonies were counted using a microscope. A colony was defined as an aggregate of 50 or more cells.

### RNA purification, reverse transcription, and real-time PCR amplification

RNA was purified using Trizol (Life Technologies), according to the manufacturer’s instructions. RNA (1 μg) was reverse-transcribed using the High-Capacity cDNA Reverse Transcription Kit (Life Technologies). cDNA (5 ng) was used for real-time PCR amplification, using commercially available Taqman probes for human *18S*, *GAPDH*, *SLC2A3*, *SLC2A1*, *SLC2A4*, *SLC2A12*, *VIM*, *ZEB1*, *SNAI1*, and *CDH1* (Life Technologies). Data were normalized to *GAPDH* or *18S* levels. For measurement of mature miR-200b, reverse transcription was performed with the TaqMan MicroRNA Reverse Transcription Kit (Life Technologies), according to the manufacturer’s instructions, separately for *miR-200b* and the normalization control, *RNU24*. cDNA (0.9 ng) was used for real-time PCR amplification.

### Immunocytochemistry

Cells (4 × 10^7^) were fixed in 4% paraformaldehyde, embedded in paraffin, and mounted on slides. Following antigen retrieval with 10 mM Na-citrate and blocking, cells were stained with anti-GLUT3 or anti-E-cadherin antibodies overnight at 4°C, followed by washing and staining with biotin-conjugated secondary antibodies for 1 h at room temperature. After washing, avidin-biotin horseradish peroxidase complexes were added for 30 min (ABC kit, Vectastain), and the complexes were revealed with a DAB peroxidase substrate kit (Vector Laboratories, Burlingame, CA, USA). Counterstain was performed using Harris hematoxylin.

### Reagents

Recombinant human TGF-β2 was from PeproTech (Rocky Hill, NJ, USA). Antibodies used were anti-GLUT3 (#400062, Calbiochem, San Diego, CA, USA), anti-E-cadherin (#3195, Cell Signaling Technology, Danvers, MA, USA), anti-vimentin (#5741, Cell Signaling), anti-SNAIL (#3879, Cell Signaling), anti-ZEB1 (#sc-25388, Santa Cruz Biotechnology, Inc., Dallas, TX, USA), anti-β-tubulin (#sc-9104, Santa Cruz Biotechnology), anti-CtBP (#sc-17759, Santa Cruz Biotechnology), anti-p300 (#sc-585, Santa Cruz Biotechnology), and normal rabbit IgG (#12-370, Millipore, Billerica, MA, USA).

### Virus production and infection

293 T cells were transfected using the calcium-phosphate precipitation method, co-transfecting the lentiviral plasmid of interest in conjunction with pMD2G (VSV-G protein) and pCMVR8.74 (lentivirus packaging vector, kind gift of D. Trono, EPFL, Lausanne). Viral supernatants were harvested 24 and 36 h post-transfection, filtered, and used directly for infection of cell lines, as described. Puromycin selection was performed to select cells with stable pRDI292-CMV (delivering control, human *GLUT3*, mouse *Snail*, or mouse *Zeb1* cDNA) or pLKO.1 (with control or *GLUT3* shRNA) genomic integration. Fluorescence-activated cell sorting was used to select cells with stable pSicoR constructs, based on GFP expression.

### Statistics

Unless specified differently, *P* values were determined by Student’s *t* tests.

### ChIP-seq analysis

Publicly available replicate ChIP-seq data for ZEB1 (GSM803411), RNA polymerase II (GSM803485), H3K27ac (GSM733771), as well as naked DNA (Input) (GSM733742) [16] were re-analyzed using Bowtie 2 [17] for mapping to the human GRCh37 genome with the parameters ‘--very-sensitive -M 10 -p 8’ and CCAT 3.0 [18] for peak-calling using the parameters ‘fragmentSize 200 slidingWinSize 100 movingStep 50 isStrandSensitiveMode 1 minCount 13 outputNum 100000 randomSeed 123456 minScore 5 bootstrapPass 80’. Data were visualized using IGV Browser [19].

### ChIP-PCR

ChIP was performed as described previously [20], using 6 × 10^7^ SW1573 and 5 μg of each antibody. To amplify regions within the promoter region of *SLC2A3*, the following primers were used: 5′-ACTGCCCTGATAGTTGGTCTGG-3′ with 5′-TTTGCCAGTGTTCCTTTCTTCG-3′ (−608 and −523 bp upstream of *SLC2A3* TSS); 5′-ACTGCCCTGATAGTTGGTCTGG-3′ with 5′-GAGGGAAAGACAGCCTGAGAGA-3′ (−608 and −482 bp upstream of *SLC2A3* TSS). To amplify regions from the intron 2, the following primers were used: 5′-CATCACAGTTGCTACAATCGGC-3′ with 5′-ACCATGCCTGGCCTTAAATTCT-3′ (2,396 and 2,559 bp downstream of *SLC2A3* TSS); 5′-ACCATGCCTGGCCTTAAATTCT-3′ with 5′-AGCCTCAGGAGTAGCTGGGACT-3′ (2,452 and 2,594 bp downstream of *SLC2A3* TSS); 5′-GTGAGTGCCAGGCCACAATAAT-3′ with 5′-TGTGTTGCTCAGGATGGTGTTT-3′ (2,466 and 2,647 bp downstream of *SLC2A3* TSS); 5′-AGTCCCAGCTACTCCTGAGGCT-3′ with 5′-TTCCGGGAGTAAGTGAGCTTTG-3′ (2,573 and 2,791 bp downstream of *SLC2A3* TSS); 5′-AAACACCATCCTGAGCAACACA-3′ with 5′-TGAGGTGAAAGAGTGGGAGGAA-3′ (2,626 and 2,831 bp downstream of *SLC2A3* TSS); 5′-TTCCTCCCACTCTTTCACCTCA-3′ with 5′-GCACCGATGTTCACAGTCTACC-3′ (2,810 and 2,926 bp downstream of *SLC2A3* TSS); 5′-AAGCTGGGTTCCCTTAGCAGAG-3′ with 5′-AAAGGGTTGGTGGAAGAACAGA-3′ (2,877 and 3,117 bp downstream of *SLC2A3* TSS); 5′-CAGTCTGTTCTTCCACCAACCC-3′ with 5′-AGGACCAGAGAGACGTGAGCAG-3′ (3,093 and 3,212 bp downstream of *SLC2A3* TSS). The intergenic region upstream of *GAPDH* was amplified, using primers 5′-ATGGGTGCCACTGGGGATCT-3′ and 5′-TGCCAAAGCCTAGGGGAAGA-3′, as described previously [21]. As positive control, a DNA sequence of *CDH1* known to bind ZEB1 was amplified using primers 5′-GGCCGGCAGGTGAACCCTCA-3′ and 5′-GGGCTGGAGTCTGAACTGA-3′, as described previously [22]. Real-time PCR was performed using SyBR green. Fold enrichment was calculated as follows: 2^^ΔΔct^ (ΔΔct = Δct^specific antibody^ − Δct^IgG^). Data were normalized using the negative control, an untranscribed region upstream of *GAPDH*, and represented as fold enrichment/fold enrichment of the negative control locus.

### Microarray analysis

We collected publicly available datasets from journal articles and Gene Expression Omnibus (GEO) repository, selecting those with medium to large sample size [23,24]. The .CEL files were imported into R/Bioconductor (http://www.bioconductor.org/) and RMA normalized [25]. A total of 462 early-stage untreated lung NSCLC samples were available, with associated patients’ overall survival. The probe IDs with higher variation for each gene were retained from a total of 13,960 genes. The normalized data together with the clinico-pathological variables were then used for further analysis. Pairwise Spearman correlations between *GLUT3* and other available genes were calculated. Univariate and multivariate survival analyses were performed using proportional Cox regression (package ‘survival’, *R*). Kaplan-Meier figure was reported showing hazard ratio (HR), 95% confidence intervals, and Wald p_value from Cox regression model for a specific comparison of interest. The continuous *GLUT3* expression was dichotomized in high and low risk levels by the median of the expression for visualization purposes. Multivariate analysis was performed with the following variables: *GLUT3* continuous expression, stage, histology, gender, and age (dichotomized by the median of 62 years).

## Results

### GLUT3 expression correlates with EMT

**Figure 1:**
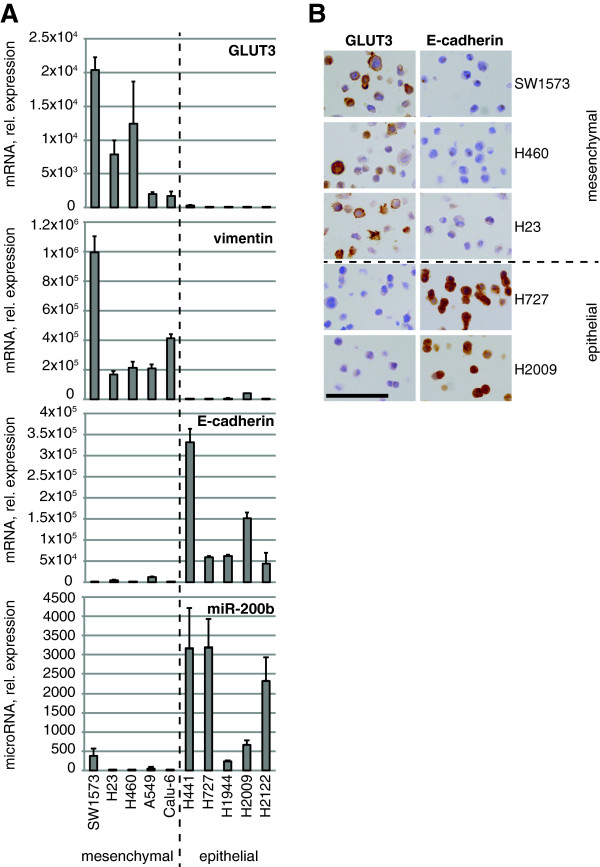
**GLUT3 is strongly expressed in mesenchymal lung tumor cells. (A)** The indicated cell lines were lysed for RNA preparation followed by reverse transcription. The cDNA was amplified by real-time PCR using probes specific for the indicated genes or internal controls. Data show means ± s.d. (*n* = 3) of mRNA or mature microRNA expression relative to the cell line expressing the least amount of the same gene (set to 1). **(B)** The indicated cells were stained to analyze the expression of GLUT3 or E-cadherin by immunocytochemistry. Scale bar, 100 μm. In **(A)** and **(B)**, the *dashed line* indicates the separation between the mesenchymal and the epithelial groups of cells.

To determine if the neuronal glucose transporter GLUT3 is expressed in lung tumor cells, we used a panel of ten human NSCLC cell lines. We noticed that *GLUT3* expression, as determined by real-time PCR analysis, varied considerably between the cell lines, with five of them expressing high and the other five expressing low *GLUT3* mRNA levels (Figure 1A). Several molecular markers can be used to classify human lung tumor cells according to their EMT status [26]. We monitored the epithelial or mesenchymal status in these cells by assessment of RNA levels of *E-cadherin* and the mature form of *miR-200b* (two markers of the epithelial state), and *vimentin* (a prototypical mesenchymal marker) using real-time PCR. These three markers clearly separated the cell lines into two groups: an ‘epithelial group’ (composed of H441, H727, H1944, H2009, and H2122 cells) that expresses *E-cadherin* and *miR-200b*, but almost no *vimentin*, and a ‘mesenchymal group’ (SW1573, H23, H460, A549, and Calu-6) that expresses high levels of *vimentin*, but very little *E-cadherin* and *miR-200b. GLUT3* expression was elevated specifically in the mesenchymal group, whereas levels were very low in cell lines of the epithelial group (Figure 1A), strongly suggesting that this glucose transporter is up-regulated during or after an EMT. To determine if the correlation with EMT was specific for GLUT3, or if other transporters within this family had a similar expression pattern, we monitored the expression of additional GLUT family members that carry glucose (GLUT1, GLUT2, GLUT4, GLUT12, and GLUT14). Whereas *GLUT2* and *GLUT14* were undetectable across the cell lines (MM and EM, unpublished observations), *GLUT1*, *GLUT4*, and *GLUT12* were expressed, but none of them showed any correlation with EMT (Additional file 1). Next, to confirm that high *GLUT3* mRNA expression in mesenchymal cells reflected actual protein expression, we performed immunocytochemistry analyses. This demonstrated that GLUT3 protein was expressed and localized to the plasma membrane of mesenchymal cells, but was not detectable in the cells from the epithelial group (Figure 1B); once again, there was an inverse correlation between GLUT3 and E-cadherin expression.

### GLUT3 is induced during EMT

**Figure 2:**
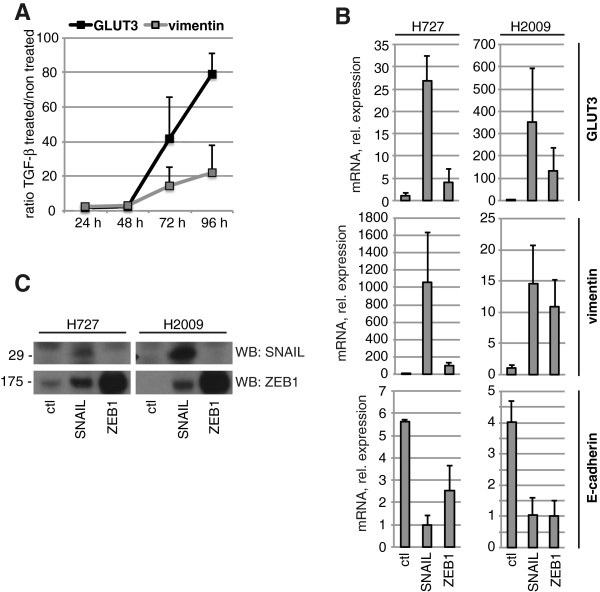
**GLUT3 is induced upon EMT. (A)** H2122 cells were stimulated with 10 ng/ml TGF-β for the indicated time points, after which the cells were lysed for RNA preparation followed by reverse transcription. The cDNA was amplified by real-time PCR using probes specific for the indicated genes or internal controls. Data show means ± s.d. (*n* = 4) of mRNA expression ratios between TGF-β treated and non-treated conditions. **(B)** H727 or H2009 cell populations stably expressing a control plasmid (ctl), SNAIL, or ZEB1 were lysed for RNA preparation followed by reverse transcription. The cDNA was amplified by real-time PCR using probes specific for the indicated genes or internal controls. Data show means ± s.d. (*n* = 3) of mRNA expression relative to the cell line expressing the least amount of the same gene (set to 1). **(C)** H727 or H2009 stable cell populations were lysed with RIPA buffer to prepare protein extracts and to analyze the expression of the indicated proteins by Western blot.

**Figure 3:**
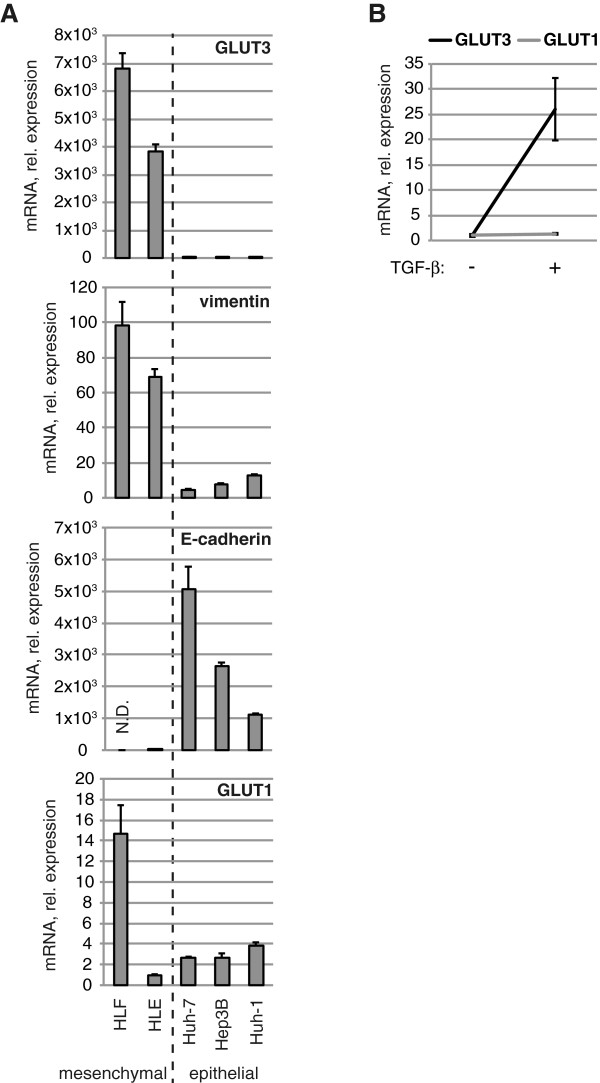
**GLUT3 is strongly expressed in mesenchymal liver tumor cells. (A)** The indicated cell lines were lysed for RNA preparation followed by reverse transcription. The cDNA was amplified by real-time PCR using probes specific for the indicated genes or internal controls. Data show means ± s.d. (*n* = 3) of mRNA expression relative to the cell line expressing the least amount of the same gene (set to 1). *N.D.*, not detected. **(B)** Hep3B cells were stimulated with 10 ng/ml TGF-β for 72 h, after which the cells were lysed for RNA preparation followed by reverse transcription. The cDNA was amplified by real-time PCR using probes specific for the indicated genes or internal controls. Data show means ± s.d. (*n* = 3) of mRNA expression relative to the untreated conditions (set to 1).

To test the hypothesis that *GLUT3* is induced upon EMT, we first stimulated the H2122 epithelial cell line with TGF-β, a potent EMT-inducing factor. As expected, TGF-β treatment up-regulated the expression of the mesenchymal marker *vimentin*. More importantly, *GLUT3* was concomitantly up-regulated in a temporal manner (Figure 2A). Because TGF-β is pleiotropic [27], we then directly tested the role of two known EMT-inducing transcription factors, SNAIL and ZEB1, in *GLUT3* regulation. We transduced two low-GLUT3 expressing epithelial cell lines, H727 and H2009, with lentiviruses to generate cells stably expressing either mouse SNAIL or ZEB1 protein. As anticipated, each of H727-SNAIL, H727-ZEB1, H2009-SNAIL, and H2009-ZEB1 stable cell populations showed a downregulation of *E-cadherin* and an up-regulation of *vimentin*. Furthermore, there was a 5- to 27-fold, and a 132- to 354-fold up-regulation of *GLUT3* in H727 and H2009 stable cells, respectively, compared to cells expressing a control plasmid (Figure 2B). Interestingly, and in agreement with previous findings [28], stable expression of SNAIL led to induction of endogenous ZEB1 (the reciprocal did not occur), raising the possibility that mouse SNAIL induces *GLUT3* expression indirectly through ZEB1 (Figure 2C). Moreover, the extent of *ZEB1* induction correlated to that of *GLUT3*: in H727 cells, mouse SNAIL overexpression resulted in a 2.5- and 27-fold induction of human *ZEB1* and *GLUT3* mRNA, respectively, whereas it was 9- and 354-fold in H2009 cells (Additional file 2). Next, to assess if the link between GLUT3 and EMT is specific for NSCLC, or whether it occurs in other tumor types, we analyzed *GLUT3* expression in human cell lines derived from HCC. Based on marked differences in the expression of *vimentin* and *E-cadherin*, we could distinguish two groups of cells: an epithelial group (Huh-7, Hep3B, and Huh-1) and a mesenchymal group (HLF and HLE). Similar to NSCLC cell lines, *GLUT3* but not *GLUT1* expression was specifically elevated in the mesenchymal group, and very low in the epithelial group (Figure 3A). Additionally, TGF-β treatment of the low-*GLUT3* expressing cell line, Hep3B, led to increased expression of *GLUT3*, whereas *GLUT1* levels remained unchanged (Figure 3B). Altogether, these data demonstrate that the regulation of GLUT3 during EMT occurs in at least two different cancer types, NSCLC and HCC.

### ZEB1 induces GLUT3 through direct binding and transcriptional activation

**Figure 4:**
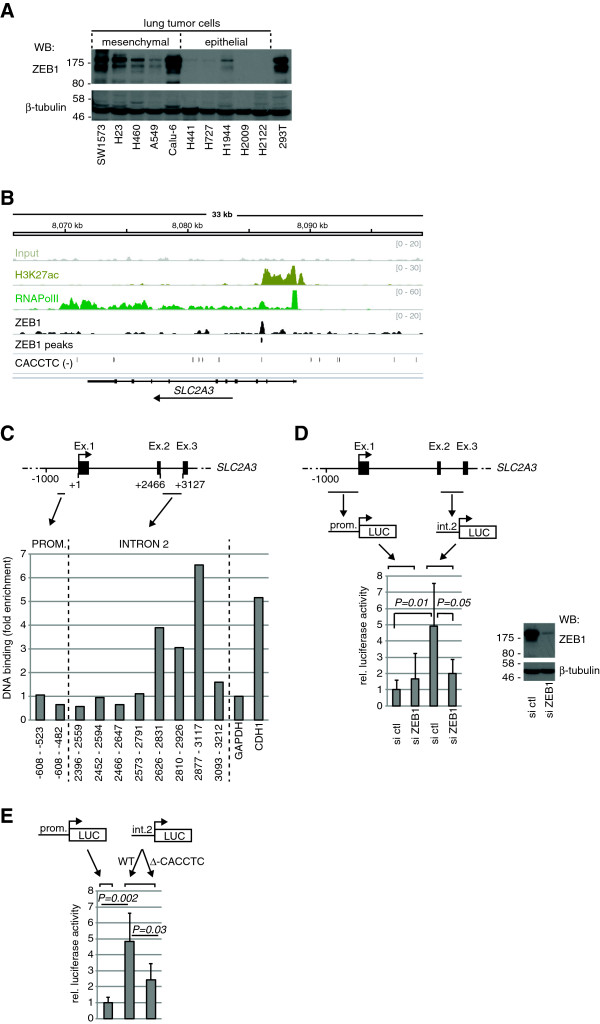
**GLUT3 is a direct ZEB1-target gene. (A)** The indicated cell lines were lysed with RIPA buffer to prepare protein extracts and to analyze the expression of ZEB1, or β-tubulin as control, by Western blot. **(B)** IGV Browser view of 33 kb around the *SLC2A3* gene including ZEB1 (*black*), RNA Polymerase II (RNAPolII, *green*), the active histone mark H3K27ac (*green*-*yellow*) ChIP-seq, and Input (*grey*) tracks in human lymphoblastoid cell lines. ZEB1 peak calls and ‘CACCTC’ sequence matches on the reverse strand are also indicated (*black*). The orientation of *SLC2A3* is indicated by an *arrow*. **(C)** Extracts from SW1573 cells were used for ZEB1 ChIP followed by real-time PCR. The coordinates of the PCR amplicons relative to the TSS are indicated. *CDH1* and an untranscribed sequence upstream of *GAPDH* were used as positive and negative controls for ZEB1 binding, respectively. The data shown are representative of three independent experiments. **(D)** (*Upper part*) scheme showing the promoter region of 1,000 bp preceding the transcription start site (prom.) and the intron 2 region (int.2), which were amplified from the *SLC2A3* gene and cloned into the luciferase reporter construct. *Ex.* exon, *LUC* luciferase. (*Lower part*) 293 T cells were transfected with the indicated luciferase reporter plasmids. Luciferase activity was analyzed 24 h later. Data show means ± s.d. (*n* = 5) of relative luciferase activity. (*Lower right*) 293 T cells were transfected with control (ctl) or ZEB1 siRNAs and were lysed 96 h later to prepare protein extracts and to analyze the expression of ZEB1, or β-tubulin as control, by Western blot. **(E)** 293 T cells were transfected with the indicated luciferase reporter plasmids. For the int.2 construct, either wild-type (WT) or a variant with a deletion of the E-box-like motif (Δ-CACCTC) was used. Luciferase activity was analyzed 24 h later. Data show means ± s.d. (*n* = 5) of relative luciferase activity.

Although ZEB1 is best characterized as a transcriptional repressor, depending on co-factor recruitment—and probably other parameters—it can activate transcription. For example, ZEB1 represses gene transcription when bound to the co-repressor C-terminal-binding protein (CtBP), whereas it activates target gene expression when bound to p300, P300/CBP-associated factor (P/CAF), or receptor-regulated (R)-SMADs [29-32]. Co-immunoprecipitation experiments performed on extracts from mesenchymal lung tumor cells revealed endogenous interactions between ZEB1 and CtBP, as well as ZEB1 and p300, suggesting ZEB1 forms different complexes to trigger target gene repression or activation in these cells (Additional file 3). To test the hypothesis that ZEB1 directly activates *GLUT3* (*SLC2A3*) gene transcription, ZEB1 mRNA and protein expression was first monitored in the panel of lung tumor cells. This showed that ZEB1 was more strongly expressed in GLUT3-proficient, mesenchymal cells compared to cells from the epithelial group (Figure 4A and Additional file 1). Additionally, in response to TGF-β stimulation (see Figure 2A), there was an up-regulation of *ZEB1*, but not *SNAIL*, which preceded that of *GLUT3* (Additional file 4), suggesting *ZEB1* induction enables the activation of *GLUT3* transcription. Next, we analyzed public data from lymphoblastoid cell extracts subjected to chromatin immunoprecipitation using anti-ZEB1, anti-RNA polymerase II or anti-histone H3 (acetyl K27) antibodies, followed by DNA sequencing (ChIP-seq) [16]. *SLC2A3* was actively transcribed in these cells, as inferred from the occupancy of RNA polymerase II and the acetylation of histone H3 on Lysine 27 at the *SLC2A3* locus (Figure 4B). Importantly, an interaction between ZEB1 and a region located within the second intron of *SLC2A3* was uncovered, with a peak of interaction that localized to an E-box-like motif, composed of a CACCTC sequence (Figure 4B). Of note, ZEB1 has been reported to bind such DNA sequences to regulate transcription [33,34]. To determine if a similar ZEB1-*SLC2A3* gene interaction occurred in GLUT3-expressing lung tumor cells, we performed ChIP-PCR analyses in extracts of SW1573 cells. As positive and negative controls, respectively, we amplified a sequence of *E-cadherin* (*CDH1*) known to bind ZEB1 and an intergenic sequence upstream of *GAPDH*[21,22]. ZEB1 bound to DNA sequences located within intron 2 of *SLC2A3*, but not to sequences from the promoter region (Figure 4C). Having established the localization of ZEB1 binding to the *SLC2A3* gene, we cloned the DNA sequence of intron 2 or, for comparison, a 1-kb sequence preceding the *SLC2A3* transcription start site into a luciferase reporter plasmid (Figure 4D). Upon transfection into 293 T cells, which express high amounts of endogenous ZEB1 (see Figure 4A), the basal luciferase activity was significantly stronger from the intron 2 construct than from the promoter construct. Also, luciferase activity from the intron 2 construct significantly decreased upon *ZEB1* knockdown (Figure 4D). Furthermore, deletion of the E-box-like motif in intron 2 substantially reduced luciferase activity, revealing the CACCTC sequence as an important functional element in response to ZEB1 (Figure 4E). Collectively, these results demonstrate that GLUT3 is induced during EMT by a mechanism involving direct binding and transcriptional activation by ZEB1.

### GLUT3 promotes glucose uptake and proliferation of mesenchymal lung tumor cells

**Figure 5:**
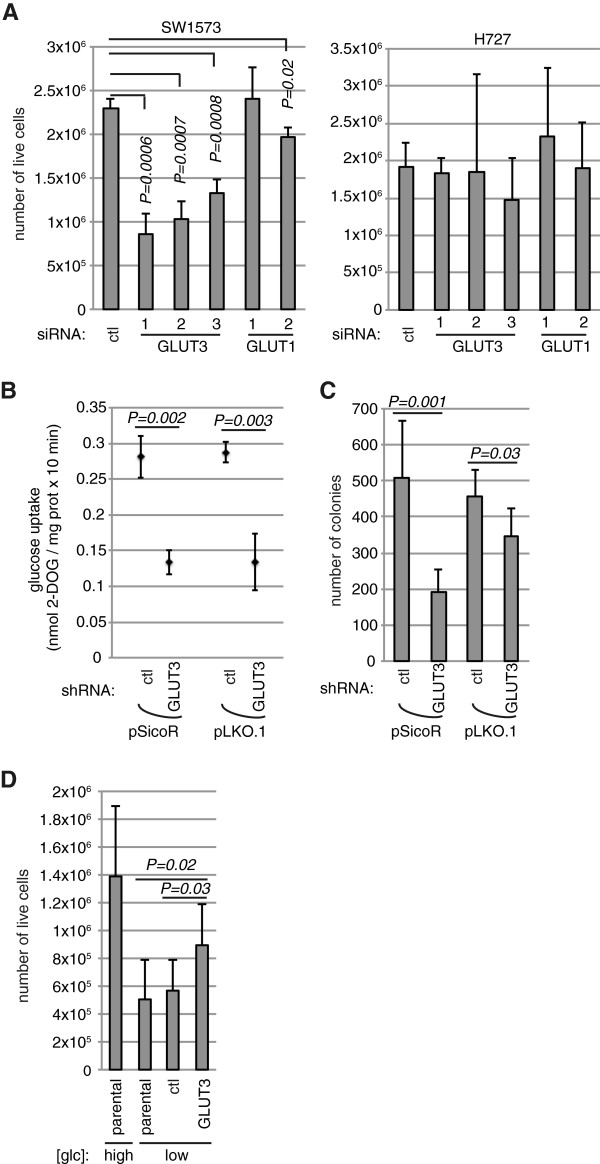
**GLUT3 promotes glucose uptake and the proliferation of mesenchymal lung tumor cells. (A)** Mesenchymal (SW1573) or epithelial (H727) cells were transfected with control (ctl) siRNA, each of three different siRNAs to decrease *GLUT3*, or each of two siRNAs to decrease *GLUT1*, as indicated. One hundred forty-four hours later, live cells were counted by trypan blue exclusion (*n* = 3 or 4). **(B)** 2-Deoxy-d-[^3^H]glucose (DOG) incorporation was measured in SW1573 cells stably expressing ctl shRNAs or shRNAs targeting *GLUT3* (in pSicoR or pLKO.1 vectors, see ‘Methods’). Data show means ± s.d. (*n* = 3) of glucose uptake (nmol) measured for 10 min, normalized to protein concentration. **(C)** SW1573 cells stably expressing ctl shRNAs or shRNAs targeting *GLUT3* (in pSicoR or pLKO.1 vectors) were prepared in soft agar for anchorage-independent growth. Three weeks later, the number of colonies was determined (*n* = 6). **(D)** H727 cells, either parental or stably expressing a control (ctl) plasmid or a *GLUT3* cDNA, were cultured in high or low glucose (glc) concentrations for 4 days, after which live cells were counted by trypan blue exclusion (*n* = 7).

We next sought to determine the functional consequences of high GLUT3 expression in mesenchymal lung tumor cells using *GLUT3* knockdown. Transfection of small interfering (si)RNAs to *GLUT3* into SW1573 cells caused a reduction in the number of live cells, whereas *GLUT1* knockdown did not or only slightly reduced cell number (Figure 5A and Additional file 5). The proportion of dead cells was not changed upon *GLUT3* knockdown in SW1573 cells (Additional file 5), suggesting that GLUT3 primarily regulates proliferation of these cells, not survival. In contrast, H727, a low-GLUT3 expressor, was not affected by *GLUT3* knockdown. Next, we transduced SW1573 cells with lentiviruses delivering short hairpin (sh)RNAs targeting *GLUT3* to generate cells with stable *GLUT3* knockdown (Additional file 5). We first used these cells to interrogate the contribution of GLUT3 to glucose import. Knockdown of *GLUT3* with two independent shRNAs led to a decrease of more than 50% in the efficiency of radioactive 2-deoxyglucose incorporation, revealing that GLUT3 plays a prominent role in glucose uptake of mesenchymal cells (Figure 5B). Importantly, *GLUT3* knockdown also led to a reduction in the number of colonies grown in anchorage-independent conditions (Figure 5C). Next, to determine if *GLUT3* knockdown had an impact on the mesenchymal state of tumor cells, we monitored *vimentin* and *E-cadherin* expression. Interestingly, *vimentin* expression was substantially decreased upon *GLUT3* knockdown (Additional file 6). Hence, although we did not observe any statistically significant variation in *E-cadherin* mRNA levels, these data suggest that GLUT3-dependent glucose uptake participates in the maintenance of a mesenchymal state. Finally, we wanted to determine if increased GLUT3 expression is sufficient to impact on the growth of lung tumor cells with epithelial traits. To do this, we generated H727 cells stably expressing human GLUT3 (Additional file 7). In high glucose concentrations, there was no difference in the proliferation of cells overexpressing GLUT3 and control cells (Additional file 7). However, we reasoned that because parental H727 cells have adapted to grow in high glucose levels without GLUT3, ectopic expression of this high-affinity glucose transporter might affect proliferation specifically when glucose concentrations are low. Indeed, the growth rate of parental cells was diminished by a reduction in glucose levels. Under these conditions, H727-GLUT3 cells grew faster than control cells, demonstrating that GLUT3 expression is sufficient to increase cancer cell proliferation in conditions where glucose concentrations are limiting (Figure 5D). Altogether, these results demonstrate an important contribution for GLUT3 in lung tumor cell proliferation.

### GLUT3 expression in human NSCLC correlates with poor overall survival and EMT signatures

**Figure 6:**
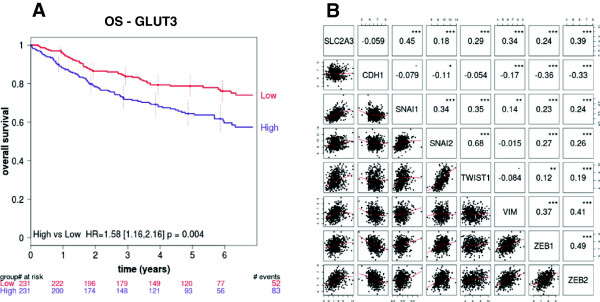
**GLUT3 expression correlates with poor overall survival and EMT in human NSCLC*. *(A)** Kaplan-Meier plot representing overall survival comparing tumor samples with high or low *GLUT3* levels. The numbers of living patients are indicated for each year and group. *HR* hazard ratio, *OS* overall survival. **(B)** The correlations between each of *GLUT3* (*SLC2A3*), *E-cadherin* (*CDH1*), *SNAIL* (*SNAI1*), *SLUG* (*SNAI2*), *Twist* (*TWIST1*), *vimentin* (*VIM*), *ZEB1*, and *ZEB2* were assessed from the pooled dataset. Spearman correlation coefficients (*indicated numbers*) were calculated between the expression values of each pair. 0.1 < *P* < 0.05; *P < 0.05; **P < 0.01; ***P < 0.001.

To assess the relevance of our findings *in vivo*, we used a combination of five publicly available microarray datasets of human NSCLC. This yielded 462 samples with follow-up of overall survival. The analysis revealed that high *GLUT3* expression was associated with a poor overall survival (Figure 6A), a finding consistent with previous studies [10,35]. Additionally, a multivariate analysis provided evidence for high *GLUT3* expression as an independent predictor of poor overall survival (Table 1). Next, we made pairwise comparisons between each of *SLC2A3*, *CDH1*, and various EMT markers or inducers (*VIM*, *SNAI1*, *SNAI2*, *ZEB1*, *ZEB2*, and *TWIST1*). As expected, most mesenchymal genes had a positive correlation with each other and a negative correlation with *CDH1*. Importantly, all mesenchymal genes showed a statistically significant positive correlation with *SLC2A3* expression (*P* < 0.001) (Figure 6B). *SLC2A3* expression was also negatively correlated with *CDH1*, as expected, although these data were not significant (Figure 6B). Altogether, these *in vivo* findings support the *in vitro* data that position GLUT3 as an important factor in the process of EMT and lung tumor progression.

**Table 1:** Univariate and multivariate analyses of overall survival in NSCLC

	**HR**	**Lower 0.95**	**Upper 0.95**	***P *** **value (Wald)**
Univariate				
SLC2A3 (continuous)	1.224	1.047	1.432	1.14E − 02
Stage (II vs. I)	1.590	1.070	2.363	2.19E − 02
Histology LCC vs. AD	1.131	0.458	2.792	7.89E − 01
Histology SCC vs. AD	1.624	1.150	2.293	5.91E − 03
Gender (F vs. M)	0.610	0.429	0.868	6.06E − 03
Age (>62 vs. ≤62)	1.906	1.385	2.622	7.47E − 05
Multivariate				
SLC2A3 (continuous)	1.194	1.016	1.404	3.16E − 02
Stage (II vs. I)	1.432	0.945	2.170	9.04E − 02
Histology LCC vs. AD	1.006	0.406	2.495	9.90E − 01
Histology SCC vs. AD	1.552	1.071	2.248	2.03E − 02
Gender (F vs. M)	0.702	0.484	1.020	6.33E − 02
Age (>62 vs. ≤62)	1.829	1.323	2.528	2.56E − 04

*AD* adenocarcinoma, *F* female, *HR* hazard ratio, *LCC* large cell carcinoma, *M* male, *SCC* squamous cell carcinoma.

## Discussion

In this study, we have identified a unique role for glucose transporter GLUT3 in the proliferation of lung tumor cells with mesenchymal characteristics. We found that GLUT3 is strongly up-regulated during EMT and contributes to glucose uptake specifically in mesenchymal-like lung tumor cells. Furthermore, GLUT3 overexpression is sufficient to substantially enhance the proliferation of lung tumor cells with epithelial traits specifically in the context of low glucose. Interestingly, increased glucose consumption was shown to promote EMT via stabilization of SNAIL [36], suggesting that increased expression of GLUT family members promotes the induction and/or maintenance of EMT. In our experiments, we did not detect an EMT occurring in response to ectopic GLUT3 protein expression in epithelial cells (MM and EM, unpublished observations), but we found a decrease in *vimentin* expression upon *GLUT3* knockdown in mesenchymal cells. Collectively, these data suggest the intriguing possibility that GLUT3 plays a role in the successful establishment or maintenance of the EMT, when tumor cells become more motile and invasive, or in tumor cell survival upon loss of cell-cell adhesion.

EMT in cancer is a complex phenomenon that is orchestrated by different transcription factors with partially overlapping gene targets. These transcription factors, like SNAIL or ZEB1, often act as transcriptional repressors. However, the mechanisms of target gene regulation are not fully elucidated. For example, ZEB1 inhibits the transcription of target genes when bound to CtBP [29,31]. In contrast, ZEB1 binding to R-SMADs and the acetyltransferases p300 and P/CAF promotes target gene transcription [30,32]. Likewise, in our study, we have demonstrated that ZEB1 can interact with each of CtBP and p300 in mesenchymal lung tumor cells and that it is a direct activator of *SLC2A3* gene transcription. Of note, ZEB1 expression was higher in the mesenchymal, GLUT3-expressing cell lines compared to the epithelial cell lines, and *ZEB1* was induced prior to *GLUT3* in response to TGF-β (see Figures 2A and 4A, and Additional files 1 and 4). This is in agreement with a previous finding reporting that the *SLC2A3* gene was one of the top 50 genes correlating positively with *ZEB1* gene expression in a panel of human lung tumor cell lines [37]. In the future, it will be important to decipher all of the genes that are directly targeted—either repressed or induced—by ZEB1 in lung cancer and to determine if this includes additional genes that regulate tumor cell metabolism.

The increased GLUT3 expression observed in tumor cells upon EMT may have important implications for cancer treatment. Indeed, not only is EMT well recognized as a crucial process that increases invasive capacities of tumor cells and promotes the acquisition of stem cell characteristics [38], it may occur earlier in tumor development than previously anticipated. For example, in a *K-ras*^*LSL-G12D/+*^; *p53*^*Flox/Flox*^ mouse model of PDAC, EMT and invasiveness precede the detection of tumors *in situ*[39]. Interestingly, a molecular classification of human PDAC was performed recently, where three subtypes were defined: classical, exocrine-like, and quasi-mesenchymal (which has the worst prognosis of all three) [40]; *SLC2A3* was one of a 20-gene signature of the quasi-mesenchymal subtype. This observation, together with our findings of a connection between GLUT3 and EMT in human liver cells from HCC, indicates that the regulation of GLUT3 by EMT extends beyond lung cancer.

## Conclusions

By uncovering direct regulation of *GLUT3* by the transcription factor ZEB1, our study provides evidence for a tight association between two central characteristics of carcinoma development: EMT and glucose metabolism. Because GLUT3 expression is mainly restricted to the brain of healthy individuals, the future development of small molecule compounds that selectively block GLUT3 and that do not cross the blood-brain barrier may become a viable strategy to treat patients with NSCLC and possibly other cancer types.

## Abbreviations

EMT: Epithelial-mesenchymal transition; HCC: Hepatocellular carcinoma; NSCLC: Non-small cell lung cancer; PDAC: Pancreatic ductal adenocarcinoma; SLC2A3: Solute carrier family 2 (facilitated glucose transporter), member 3; ZEB1: Zinc finger E-box-binding homeobox 1.

## Competing interests

The authors declare that they have no competing interests.

## Authors’ contributions

MM participated in the design of the study, performed most of the experiments, and analyzed the data. JV performed several experiments and analyzed the data. SR did the bioinformatics analyses of the microarray datasets. SG carried out the immunoprecipitation experiments and analyzed the data. NS carried out the ZEB1 knockdown experiments and analyzed the data. PCS participated in the bioinformatics analyses of the ChIP-seq datasets. BD participated in the bioinformatics analyses of the ChIP-seq datasets. LEF carried out the initial real-time PCR experiments of the cell line panel and analyzed the data. JG, DM, and TGO analyzed the data. EM conceived the study, participated in its design and coordination, analyzed the data, and wrote the manuscript. All authors read and approved the final manuscript.

## Supplementary Material

Additional file 1: Figure S1 GLUT1, GLUT4, GLUT12, and ZEB1 expression in mesenchymal and epithelial lung tumor cells. The indicated cell lines were lysed for RNA preparation followed by reverse transcription. The cDNA was amplified by real-time PCR using probes specific for the indicated genes or internal controls. Data show means ± s.d. (*n* = 3) of mRNA expression relative to the cell line expressing the least amount of the same gene (set to 1). When there was no specific amplification after 40 cycles of PCR, the samples were called not determined (N.D.). Click here for file

Additional file 2: Figure S2 hZEB1 and hGLUT3 induction upon mSNAIL overexpression. H727 or H2009 cell populations stably expressing a control plasmid (ctl) or mouse (m) SNAIL were lysed for RNA preparation followed by reverse transcription. The cDNA was amplified by real-time PCR using probes specific for human *ZEB1*, *GLUT3*, or an internal control. Data show means ± s.d. (*n* = 3) of mRNA expression, represented as fold induction (mSNAIL/ctl) for each cell line. The arrows and numbers indicate the fold induction between samples. Click here for file

Additional file 3: Figure S3 ZEB1 interacts with CtBP and p300 in mesenchymal lung tumor cells. Protein extracts from the indicated cell lines were used for ZEB1 or control (IgG) immunoprecipitation, followed by Western blot using the indicated antibodies. The arrowheads indicate the position of each protein. XT, cell extract; ø, empty lane. Click here for file

Additional file 4: Figure S4 *ZEB1* is induced rapidly upon TGF-β stimulation. H2122 cells were stimulated with 10 ng/ml TGF-β for the indicated time points, after which the cells were lysed for RNA preparation followed by reverse transcription. The cDNA was amplified by real-time PCR using probes specific for *ZEB1*, *SNAIL*, or an internal control. Data show means ± s.d. (*n* = 4) of mRNA expression ratios between TGF-β treated and non-treated conditions. Click here for file

Additional file 5: Figure S5 Efficiency of siRNA or shRNA knockdown. (A) Efficiency of GLUT3 or GLUT1 siRNA knockdown. SW1573 cells were transfected with one of three different siRNAs to target GLUT3, two to target GLUT1, or a control (ctl) siRNA, or were left untransfected (--). Seventy-two hours later, the cells were lysed for RNA preparation followed by reverse transcription. The cDNA was amplified by real-time PCR using probes specific for the indicated genes or GAPDH as an internal control. Data show the percentage of mRNA expression relative to the non-transfected condition (set to 100%). (B) Efficiency of GLUT3 siRNA knockdown. SW1573 cells were transfected with one of three different siRNAs to target GLUT3, a control (ctl) siRNA, or were left untransfected (--). Seventy-two hours later, the cells were lysed with RIPA buffer to prepare protein extracts and to analyze the expression of GLUT3 by Western blot. (C) GLUT3 or GLUT1 knockdown does not affect the number of dead cells. SW1573 cells were transfected with control (ctl) siRNA, each of three different siRNAs to decrease GLUT3, or each of two siRNAs to decrease GLUT1, as indicated. One hundred forty-four hours later, dead cells were counted by trypan blue exclusion (*n* = 3). (D) Efficiency of GLUT3 shRNA stable knockdown. SW1573 cells stably expressing ctl or GLUT3 shRNAs (in the indicated vectors) were lysed (left panel) with RIPA buffer to prepare protein extracts and to analyze the expression of GLUT3 by Western blot, or (right panel) for RNA preparation followed by reverse transcription. The cDNA was amplified by real-time PCR using probes specific for GLUT3 or GAPDH as an internal control. Data show the percentage of GLUT3 mRNA expression relative to the control (ctl) shRNA expressing cells (set to 100%). *n.s., non-specific. Click here for file

Additional file 6: Figure S6 *GLUT3* knockdown leads to diminished *vimentin* expression. SW1573 cells stably expressing a control (ctl) or *GLUT3* shRNA were lysed for RNA preparation followed by reverse transcription. The cDNA was amplified by real-time PCR using probes specific for the indicated genes or internal controls. Data show means ± s.d. (*n* = 3) of mRNA expression relative to the condition with the least amount of mRNA for each gene (set to 1). Click here for file

Additional file 7: Figure S7 Generation of H727 cells stably expressing GLUT3. (A) Stable expression of GLUT3. (Left) Parental H727 cells or H727 cells stably expressing a control (ctl) plasmid or a GLUT3 cDNA were lysed with RIPA buffer to prepare protein extracts and to analyze the expression of GLUT3 (or β-tubulin as loading control) by Western blot. (Right) Parental or GLUT3-expressing H727 cells were stained to analyze the expression of GLUT3 by immunocytochemistry (ICC). Scale bar, 100 μm. (B) Ectopic GLUT3 expression does not affect cell proliferation in high glucose concentrations. H727 cells, either parental or stably expressing a control (ctl) plasmid or a GLUT3 cDNA, were cultured in high glucose (glc) concentrations for 4 days, after which live cells were counted by trypan blue exclusion (*n* = 7). Click here for file
