# Supplementary figures and images for: GLUT3 is induced during epithelial-mesenchymal transition and promotes tumor cell proliferation in non-small cell lung cancer
Source: Cancer Metab. 2014 Jul 29;2:11. doi: 10.1186/2049-3002-2-11 (PMC4122054; doi:10.1186/2049-3002-2-11)

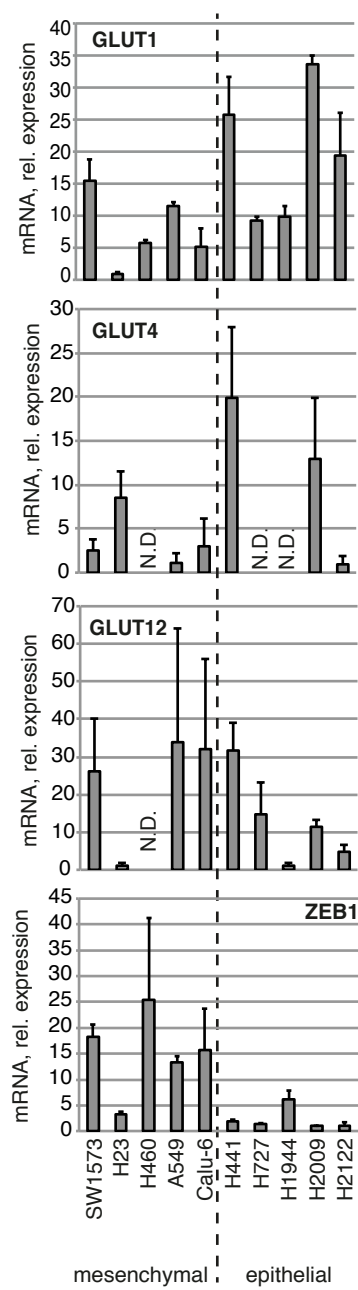

Supplement: Additional file 1: Figure S1 — GLUT1, GLUT4, GLUT12, and ZEB1 expression in mesenchymal and epithelial lung tumor cells. The indicated cell lines were lysed for RNA preparation followed by reverse transcription. The cDNA was amplified by real-time PCR using probes specific for the indicated genes or internal controls. Data show means ± s.d. (n = 3) of mRNA expression relative to the cell line expressing the least amount of the same gene (set to 1). When there was no specific amplification after 40 cycles of PCR, the samples were called not determined (N.D.). [file 2049-3002-2-11-S1.pdf]

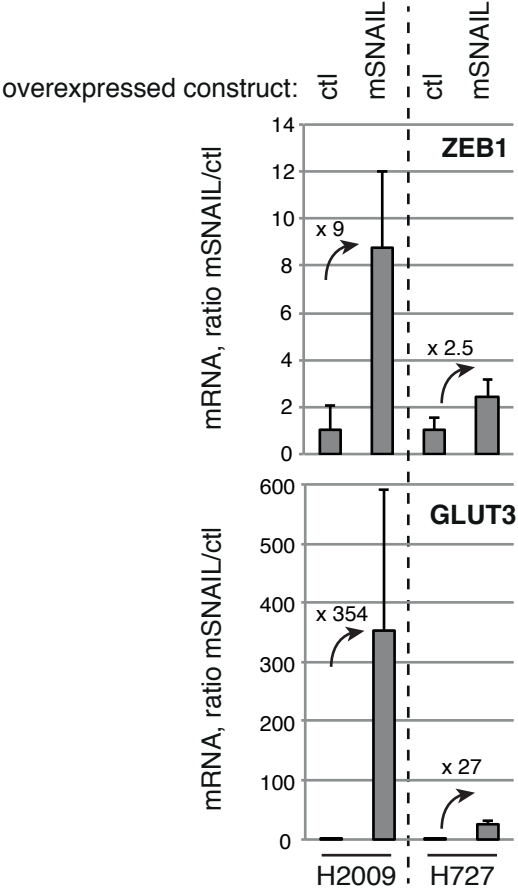

Supplement: Additional file 2: Figure S2 — hZEB1 and hGLUT3 induction upon mSNAIL overexpression. H727 or H2009 cell populations stably expressing a control plasmid (ctl) or mouse (m) SNAIL were lysed for RNA preparation followed by reverse transcription. The cDNA was amplified by real-time PCR using probes specific for human ZEB1, GLUT3, or an internal control. Data show means ± s.d. (n = 3) of mRNA expression, represented as fold induction (mSNAIL/ctl) for each cell line. The arrows and numbers indicate the fold induction between samples. [file 2049-3002-2-11-S2.pdf]

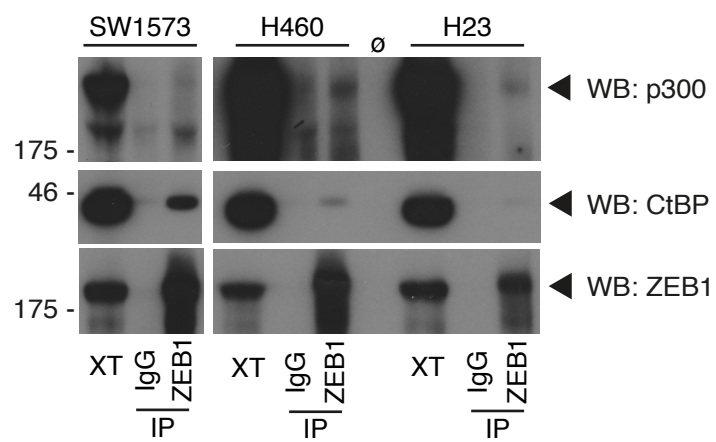

Supplement: Additional file 3: Figure S3 — ZEB1 interacts with CtBP and p300 in mesenchymal lung tumor cells. Protein extracts from the indicated cell lines were used for ZEB1 or control (IgG) immunoprecipitation, followed by Western blot using the indicated antibodies. The arrowheads indicate the position of each protein. XT, cell extract; ø, empty lane. [file 2049-3002-2-11-S3.pdf]

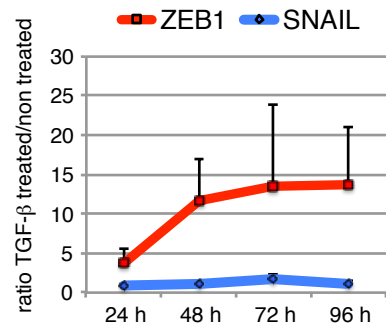

Supplement: Additional file 4: Figure S4 — ZEB1 is induced rapidly upon TGF-β stimulation. H2122 cells were stimulated with 10 ng/ml TGF-β for the indicated time points, after which the cells were lysed for RNA preparation followed by reverse transcription. The cDNA was amplified by real-time PCR using probes specific for ZEB1, SNAIL, or an internal control. Data show means ± s.d. (n = 4) of mRNA expression ratios between TGF-β treated and non-treated conditions. [file 2049-3002-2-11-S4.pdf]

**A**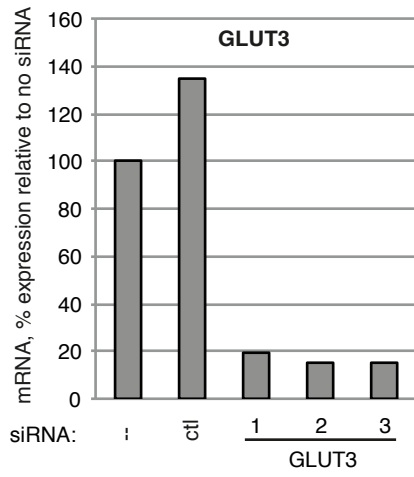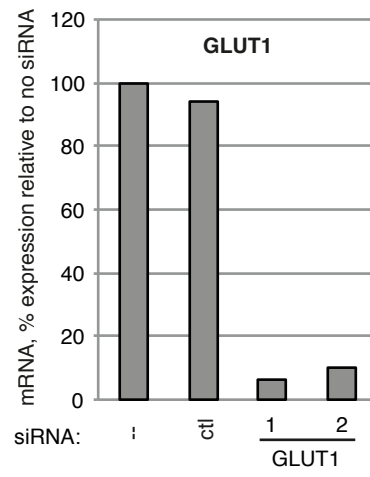**B**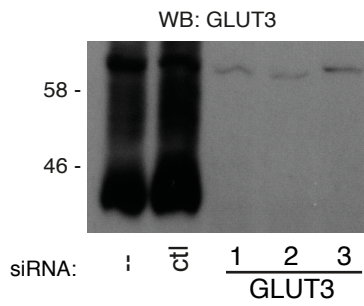**C**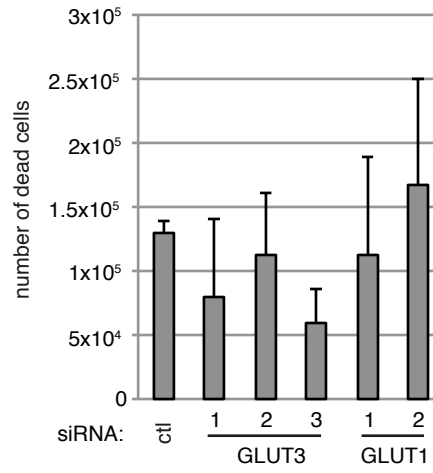**D**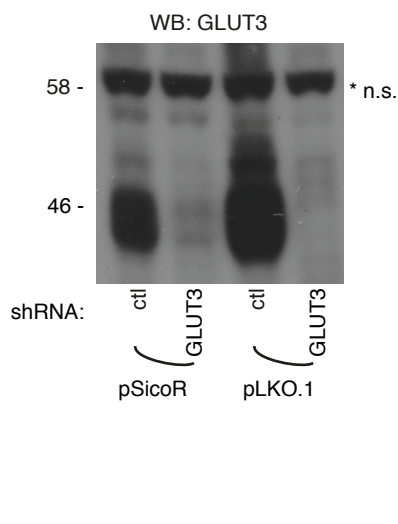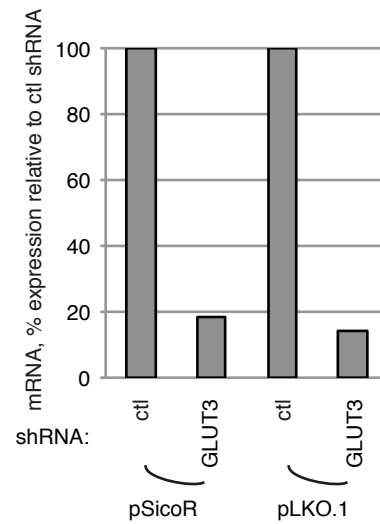

Supplement: Additional file 5: Figure S5 — Efficiency of siRNA or shRNA knockdown. (A) Efficiency of GLUT3 or GLUT1 siRNA knockdown. SW1573 cells were transfected with one of three different siRNAs to target GLUT3, two to target GLUT1, or a control (ctl) siRNA, or were left untransfected (--). Seventy-two hours later, the cells were lysed for RNA preparation followed by reverse transcription. The cDNA was amplified by real-time PCR using probes specific for the indicated genes or GAPDH as an internal control. Data show the percentage of mRNA expression relative to the non-transfected condition (set to 100%). (B) Efficiency of GLUT3 siRNA knockdown. SW1573 cells were transfected with one of three different siRNAs to target GLUT3, a control (ctl) siRNA, or were left untransfected (--). Seventy-two hours later, the cells were lysed with RIPA buffer to prepare protein extracts and to analyze the expression of GLUT3 by Western blot. (C) GLUT3 or GLUT1 knockdown does not affect the number of dead cells. SW1573 cells were transfected with control (ctl) siRNA, each of three different siRNAs to decrease GLUT3, or each of two siRNAs to decrease GLUT1, as indicated. One hundred forty-four hours later, dead cells were counted by trypan blue exclusion (n = 3). (D) Efficiency of GLUT3 shRNA stable knockdown. SW1573 cells stably expressing ctl or GLUT3 shRNAs (in the indicated vectors) were lysed (left panel) with RIPA buffer to prepare protein extracts and to analyze the expression of GLUT3 by Western blot, or (right panel) for RNA preparation followed by reverse transcription. The cDNA was amplified by real-time PCR using probes specific for GLUT3 or GAPDH as an internal control. Data show the percentage of GLUT3 mRNA expression relative to the control (ctl) shRNA expressing cells (set to 100%). *n.s., non-specific. [file 2049-3002-2-11-S5.pdf]

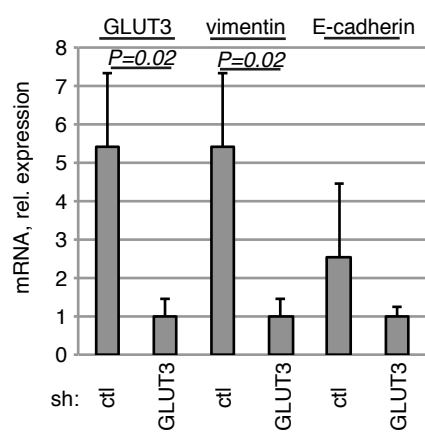

Supplement: Additional file 6: Figure S6 — GLUT3 knockdown leads to diminished vimentin expression. SW1573 cells stably expressing a control (ctl) or GLUT3 shRNA were lysed for RNA preparation followed by reverse transcription. The cDNA was amplified by real-time PCR using probes specific for the indicated genes or internal controls. Data show means ± s.d. (n = 3) of mRNA expression relative to the condition with the least amount of mRNA for each gene (set to 1). [file 2049-3002-2-11-S6.pdf]

**A**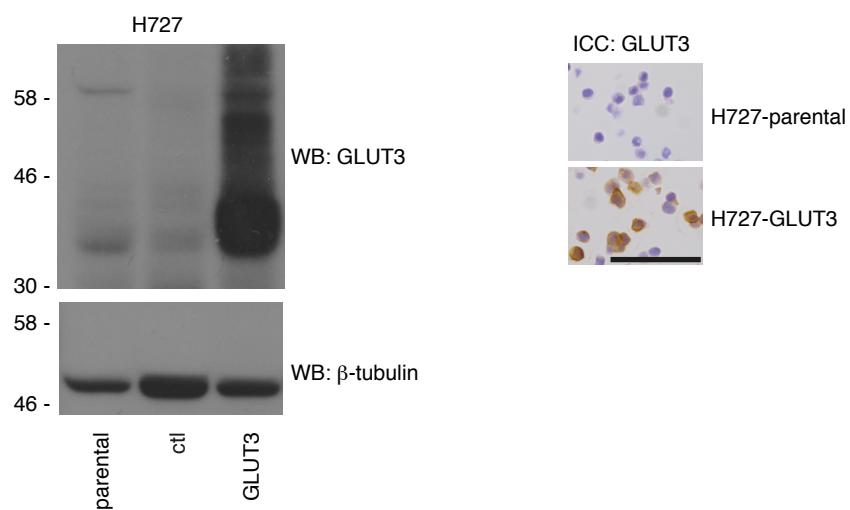**B**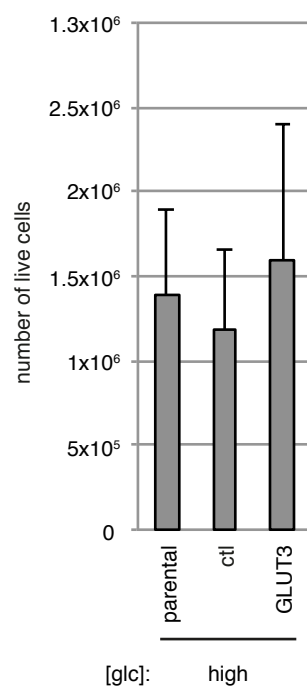

Supplement: Additional file 7: Figure S7 — Generation of H727 cells stably expressing GLUT3. (A) Stable expression of GLUT3. (Left) Parental H727 cells or H727 cells stably expressing a control (ctl) plasmid or a GLUT3 cDNA were lysed with RIPA buffer to prepare protein extracts and to analyze the expression of GLUT3 (or β-tubulin as loading control) by Western blot. (Right) Parental or GLUT3-expressing H727 cells were stained to analyze the expression of GLUT3 by immunocytochemistry (ICC). Scale bar, 100 μm. (B) Ectopic GLUT3 expression does not affect cell proliferation in high glucose concentrations. H727 cells, either parental or stably expressing a control (ctl) plasmid or a GLUT3 cDNA, were cultured in high glucose (glc) concentrations for 4 days, after which live cells were counted by trypan blue exclusion (n = 7). [file 2049-3002-2-11-S7.pdf]
